# Revolution of Chronic Lymphocytic Leukemia Therapy: the Chemo-Free Treatment Paradigm

**DOI:** 10.1007/s11912-020-0881-4

**Published:** 2020-02-05

**Authors:** Annika Scheffold, Stephan Stilgenbauer

**Affiliations:** 1 Department of Internal Medicine III, Universitätsklinikum Ulm, Albert-Einstein Allee 23, D-89081 Ulm, Germany; 2 Department of Internal Medicine I, Saarland University, D-66421 Homburg, Germany

**Keywords:** Chronic lymphocytic leukemia, BCR signaling, BTK inhibitor, Venetoclax, BCL2 inhibitors

## Abstract

**Purpose of Review:**

Over the last years, targeted anticancer therapy with small molecule inhibitors and antibodies has much replaced chemoimmunotherapy, which has been the gold standard of care for patients with chronic lymphocytic leukemia (CLL). Here we give an overview of novel targeted agents used in therapy of chronic lymphocytic leukemia, as well as efforts to overcome resistance development, focusing on approved drugs since they gained high relevance in clinical practice.

**Recent Findings:**

Novel agents moved to the forefront as a treatment strategy of CLL due to their outstanding efficacy, almost irrespectively of the underlying genetic features. Inhibition of Bruton’s tyrosine kinase (BTK), a key molecule in the B cell receptor pathway, achieved dramatic efficacy even in poor-risk and chemo-refractory patients. Further success was accomplished with venetoclax, which specifically inhibits anti-apoptotic BCL2 and induces apoptosis of CLL cells.

**Summary:**

Inhibition of BTK or BCL2 is very effective and induces prolongation of progression-free and overall survival. Approved combination treatments such as venetoclax or ibrutinib with obinutuzumab show high responses rates and long remission durations. However, evolution and selection of subclones with continuous treatment leads to resistance towards these novel drugs and disease relapse. Hence, comparison of sequential treatment with combinations and discontinuation of therapy are important aspects which need to be investigated.

## Introduction

Chronic lymphocytic leukemia (CLL) is the most common type of leukemia in adults and mainly affects the elderly [1]. CLL is a B cell malignancy, where clonal CD5^+^CD19^+^CD23^+^ B cells accumulate in peripheral blood and infiltrate secondary lymphoid organs such as lymph nodes, spleen, and bone marrow [2]. The disease is highly heterogeneous clinically mostly due to hypermutations of the immunoglobulin heavy-chain genes (IGHV), genomic aberrations, and recurrent gene mutations which associate with the clinical course [3, 4]. The mechanisms underlying CLL pathogenesis are not fully resolved. Allogenic stem cell transplantation is still the only curative therapy, although limited to a small subset of young and fit patients [5]. For the last 10 years, chemoimmunotherapy with fludarabine, cyclophosphamide, and rituximab (FCR) has been the most effective treatment but its success is limited by comorbidities, age, and fitness of the patients [6–8]. However, treatment of patients with high-risk del(17p) or *TP53* mutations have shown poor outcome [9–11].

Recent scientific advances in understanding the biology of CLL evolved in the development of novel therapeutic agents. Small molecule inhibitors targeting key survival mechanisms revolutionized therapy and showed unparalleled effects in patients irrespective of their genetic aberrations. The novel agents led to a paradigm change in patient care from treatment with unspecific DNA damaging agents to “targeted therapy.”

## Chemoimmunotherapy in CLL

The CD20 antigen is expressed on the surface of mature B cells and is one of the most successful targets in treatment of B cell malignancies. Monoclonal antibodies are widely used to deplete B cells in cancers or autoimmune diseases. The first CD20 antibody was rituximab, which has been FDA (US Food and drug administration)-approved in 1998. Several modes of action of rituximab are currently known such as complement-dependent cytotoxicity (CDC), opsonization of macrophages inducing antibody-dependent cell-mediated cytotoxicity (ADCC), and direct killing by apoptosis to a lower extent [12]. Additionally, CD20 receptor colocalizes with the B cell receptor participating in its activation and signaling. Despite that, the exact mode of action of rituximab still remains unclear. If rituximab was combined with fludarabine and cyclophosphamide, progression-free survival (PFS) and overall survival (OS) were significantly improved [13]. In the CLL8 trial, the FCR (fludarabine-cyclophosphamide-rituximab) group had a PFS of 56.8 months compared with 32.9 months in the FC (fludarabine-cyclophosphamide) arm. Here, the median OS in the FCR arm was not reached in comparison with 86 months in the FC arm [8]. IGHV-mutated patients had most benefit from FCR. However, chemoimmunotherapy is less effective with negative impact on PFS and OS in patients with unmutated IGHV, mutated *TP53* and del(17p), del(11q), and some gene mutations such as *NOTCH1*, *SF3B1*, and *BIRC3* [3, 7].

Ofatumumab is a humanized anti-CD20 monoclonal antibody which targets a different epitope than rituximab resulting in enhanced activation of CDC and similar activation of ADCC and apoptosis [14]. Ofatumumab was approved as a single agent in fludarabine refractory CLL, as well as in combination with fludarabine and cyclophosphamide for refractory CLL or with chlorambucil or bendamustine for treatment-naïve patients. Good tolerability was shown in elderly patients with a median PFS of 22.4 months (ofatumumab and chlorambucil; COMPLEMENT-1 trial) versus 13.1 months (chlorambucil monotherapy) [15]. The COMPLEMENT-2 trial demonstrated an increased PFS of relapsed patients from 18.8 months (FC) to 28.9 months (FCO) when adding ofatumumab to the FC treatment [16]. Similar to treatment with rituximab, patients with *NOTCH1* mutations benefited less. In spite of everything, the use of ofatumumab is suggested in earlier course of disease, since a phase IV study in heavily pretreated patients demonstrated limited efficacy and low numbers of responses [17].

On the contrary, obinutuzumab (GA101) is a recombinant type II anti-CD20 and immunoglobulin G1 Fc-optimized monoclonal antibody, which induces CDC and direct cell death upon binding to CD20 depending on actin reorganization and lysosome involvement [18]. A successful phase I trial showed a response rate of 62% demonstrating activity of obinutuzumab in heavily pretreated patients [19]. In the phase III CLL11 study, obinutuzumab was combined with chlorambucil (clb) and compared with rituximab-chlorambucil and chlorambucil monotherapy. Obinutuzumab-clb was superior to rituximab-clb and clb-monotherapy, more frequently associated with a negative MRD (minimal residual disease, defined as less than 1 CLL cell in 10,000 leukocytes) and prolonged PFS (median PFS 27.6 and 16.3 months vs. 11.1 months, respectively) [20]. Since obinutuzumab showed higher potency, equal tolerability and sparely enriched adverse effects than rituximab, it was FDA-approved in 2013 for untreated CLL patients in combination with chlorambucil who are not eligible for a high intensive therapy.

Infection rates of obinutuzumab were similar to rituximab, but more severe infusion-related side effects were observed. Nevertheless, rituximab is the only CD20 antibody which is broadly approved for combined use, while obinutuzumab is currently approved in combination with clb or with venetoclax (see below).

## Targeting BCR Signaling

B cell receptor (BCR) signaling is essential for CLL cells. Several novel agents target molecules of the BCR pathway and are effective even in high-risk CLL. In 2014, the FDA approved ibrutinib, an orally bioavailable BTK (Bruton’s tyrosine kinase) inhibitor, as monotherapy in relapsed/refractory CLL (R/R). The mode of action of ibrutinib operates via specific covalent binding to the cysteine 481 in the active site of the BTK enzyme. Binding to BTK inhibits downstream signaling such as MAPK, PI3K, and NF-kB, and reduces migration and proliferation of the tumor cell [21]. In patients, ibrutinib induces lymphocytosis with an asymptomatic increase of tumor cells in the peripheral blood, whereas rapid shrinkage of lymph nodes and spleen is observed. Lymphocytosis alone must not be correlated with disease progression and usually resolves within a few months of therapy [22]. Approval of ibrutinib was based on a phase Ib/II trial, where 85 heavily pretreated R/R patients reached a PFS of 75% and OS of 83% after 26 months [23•]**.** Intriguingly, the response was independent of genomic risk factors, prior therapies, or the presence of del(17p). A 5-year follow-up of an extended patient collective showed an overall response of 92% in treatment-naïve patients and 89% in R/R patients, respectively. The PFS rate of ibrutinib monotherapy was 92% in treatment-naïve patients and 44% in R/R patients, while the OS rate was 92% and 60%. Hematological adverse events (AEs) such as grade 3 cytopenia, neutropenia, and thrombocytopenia decreased over time [24].

The phase III RESONATE study compared single-agent ibrutinib and ofatumumab in high-risk relapsed patients. Follow-up after only 19 months showed a significantly longer PFS in the ibrutinib arm with 91% of patients attaining a response. The PFS at 24 month was 74%. Long-term follow-up reported a continuous response to ibrutinib, which was increasing over time. Median duration of ibrutinib was 41 months, with 46% remaining on treatment at a median follow-up of 44 months [25].

**Fig. 1:**
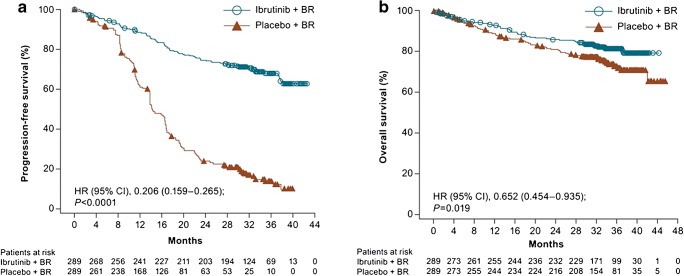
Follow-up of the phase III HELIOS trial up to 36 months. **a** Progression-free survival was not reached vs 14.3 months (*P* < 0.0001). **b** Median overall survival was not reached but significant in the ibrutinib + BR arm (*P* = 0.019). CI confidence interval, HR hazard ratio. Reprinted from Fraser G, Cramer P, Demirkan F, et al. Updated results from the phase 3 HELIOS study of ibrutinib, bendamustine, and rituximab in relapsed chronic lymphocytic leukemia/small lymphocytic lymphoma. *Leukemia*. 2019;33:969–980. 10.1038/s41375-018-0276-9, an article licensed under a Creative Commons Attribution 4.0 International License (https://creativecommons.org/licenses/by/4.0/) [26]

The phase III HELIOS trial compared ibrutinib and bendamustine with rituximab in treated CLL without del(17p). At an observation time of 18 months, BR + ibrutinib were superior with a PFS of 79% in comparison with 24% in the BR + placebo group. Impressively, the PFS at 36 months was 68% vs 13.9% (Fig. 1a). Overall survival at 36 months was 81.6% vs. 72.9%, (Fig. 1b) [26].

In the front line treatment setting, the RESONATE-2 phase III study compared ibrutinib vs. chlorambucil in elderly, untreated patients. A superior response to ibrutinib was demonstrated with a PFS of 24 months, whereas the median was not reached under treatment with chlorambucil as compared with 18.9 months under ibrutinib and an OS of 85% vs. 98.9%, respectively [27].

More recent clinical phase III trials demonstrated superiority of ibrutinib in comparison with chemoimmunotherapy (ECOG-ACRIN E1912, Alliance A041202, iILLUMINATE) [28–30].

The phase III E1912 trial demonstrated superior PFS and OS for ibrutinib + rituximab in comparison with FCR in a large cohort of 510 untreated young and fit patients without del(17p) [28]. Alliance A041202 compared ibrutinib with ibrutinib + rituximab and bendamustine + rituximab (BR). Ibrutinib monotherapy or ibrutinib + rituximab prolonged PFS in comparison with BR as a frontline regimen. Of note, the addition of rituximab did not add a benefit in comparison with ibrutinib monotherapy [29]. With iLLUMINATE, the chemotherapy free combination of ibrutinib + obinutuzumab was validated to be superior in naïve patients compared with chlorambucil + obinutuzumab [30].

Based on these extensive clinical trial data, ibrutinib is the preferred option as first line therapy in old and young patients. One disadvantage of the regimen is the need for continuous therapy, which may stimulate resistance generation due to continued selection pressure. 

Despite its initial efficacy, patients relapse under ibrutinib therapy. Interestingly, analysis of tumor cells of patients who progressed under ibrutinib therapy revealed specific mutations in BTK at position 481. Of importance, the C481S mutation of BTK conferred resistance by preventing the covalent binding of ibrutinib to its target cysteine 481 in BTK [31, 32•]. Furthermore, several mutations were identified in *PLCG2*, which functions downstream of BTK. The gain-of function mutations in *PLCG2* induce hyperreactive BCR signaling mediated by RAC2 and loss of dependence on BTK [33]. Sequencing of relapsed patient cells uncovered that 85% of all patients which relapse during ibrutinib treatment, carried a *BTK* or *PLCG2* mutation. The mutations were already detectable early after a median of 9.4 months of ibrutinib treatment. This knowledge is essential to avoid early resistance and could help to decide a possible switch of interventions [34].

## PI3K Inhibitors Idelalisib and Duvelisib

The phosphoinositide 3-kinase (PI3K) delta inhibitor idelalisib targets BCR signaling in CLL cells. It was FDA/ European Medicines Agency (EMA)-approved in 2014 for patients with refractory CLL. The approval was based on a phase III study in which idelalisib and rituximab significantly improved PFS and OS [35]. Idelalisib also showed efficacy in patients with del(17p) or TP53 mutations. Furthermore, interim results of a phase III trial demonstrated that the combination of bendamustine, rituximab, and idelalisib significantly enhanced PFS in patients with R/R CLL in comparison with BR + placebo (median PFA 20.8 vs. 11.1 months) [36]. Very recent updates of the Gilead 116/117 trial report improved efficacy of rituximab/idelalisib compared with rituximab monotherapy: an overall response rate (ORR) of 85.5% after 20.3 months of follow-up. Improved OS was the greatest among patients with del(17p) or TP53 mutation. Additionally, IGHV unmutated cases showed similar efficacy. In contrast, long-term exposure to idelalisib increased adverse effects, especially grade 3 diarrhea, colitis, and pneumonitis [37].

At the beginning of 2018, the approval of idelalisib in combination with rituximab and bendamustine for R/R CLL was withdrawn because of toxicity concerns. Black boxed warnings on idelalisib drug treatment were implemented due to fatal or serious hepatotoxicity (11–18%), %), diarrhea/colitis (14–19%), pneumonitis (4%), infections (21–36%), intestinal perforation, and transaminitis (54% grade 3) [38]. An interim analysis of three earlier-stage clinical trials demonstrated decreased overall survival in the idelalisib arms due to severe infections with PJP (pneumocystis jiroveci fungus) and CMV (cytomegalovirus). The EMA mandated additional safety measurements, PJP prophylaxis and CMV monitoring. Preclinical research data demonstrate that PI3K inhibition can modulate the effect in regulatory T cells on tumor cells, supporting the effect of PI3Kδ inhibitors. Nonetheless, CD8+ T cell function was decreased upon PI3K inhibition which might explain increased susceptibility for infections [39]. Acquired resistance to idelalisib treatment has been observed in humans, although no unique recurrent mutation was identified [40]. Preclinical data show that resistance to PI3Kδ inhibition does not rely on a unique mutation, though resistance to PI3Kδ inhibition induces a relevant activation of IGF1R, resulting in enhanced MAPK signaling [41].

Since idelalisib treatment appears to be associated with a higher rate of adverse effects than ibrutinib or venetoclax, idelalisib is not selected as the first choice for relapsed/refractory patients and remains reserved for higher lines of therapy and for patients not suitable for other therapeutic options.

Duvelisib (IPI-145) is the second FDA-approved PI3K inhibitor, targeting γ and δ isoforms. Preclinical studies demonstrate greater activity of inhibition of both isoforms γ and δ than PI3Kδ alone [42]. The phase III DUO trial compared duvelisib monotherapy with ofatumumab in patients with R/R CLL. Patients treated with duvelisib had significantly better PFS and ORR. Serious adverse effects were reported in 67% of patients treated with duvelisib. Adverse effects grade 3 occurred in 87% of the duvelisib treated patients compared with 48% in the ofatumumab-treated arm. Pneumonia, infections, and diarrhea were the most frequent [43]. Based on the DUO trial, duvelisib was FDA-approved for R/R CLL after two prior lines of therapy in 2018.

## Targeting B Cell Lymphoma 2 (BCL2)

A novel treatment principle was introduced by targeting B cell lymphoma 2 (BCL2) using the BH3 mimetics, which cause immediate apoptosis of tumor cells. BCL2 family proteins play a major role in the regulation of cell death and are highly conserved. The BCL2 family is clustered into three main functional groups, the pro-survival and anti-apoptotic proteins BCL2, MCL-1, BCL- x_L_, and BCL-w [44–46], the multi-BH domain pro-apoptotic proteins BAX and BAK, and the pro-apoptotic BH3-only proteins BIM, tBID; BAD, PUMA; NOXA, and HRK [47–49] that trigger and execute the “suicidal” cell death. In healthy cells, the balance between cell survival and cell death requires dynamic binding interactions between pro-apoptotic and anti-apoptotic proteins.

CLL cells overexpress anti-apoptotic BCL2. Overexpression of BCL2 is caused by various mechanisms. The most common cytogenetic abnormality is the del(13q14), the minimally deleted region of which includes the *BCL2* repressors and microRNAs 15 and 16 [50]. Moreover, hypomethylation of *BCL2* in CLL also contributes to *BCL2* upregulation due to epigenetic dysregulation [51, 52]. On the other hand, defects in expression of pro-apoptotic members result in a loss of the tumor suppressive function and lead to an imbalance between pro-and anti-apoptotic BCL2 family proteins. Homozygous deletions or inactivating mutations of BAX and BID [53, 54] or defective expression of BID and PUMA due to loss of p53 function also tip the balance towards anti-apoptotic proteins [55, 56].

BCL2 can be selectively targeted with venetoclax which is a novel, orally bioavailable BH3 mimetic. Venetoclax shows high efficacy in particular in the treatment of CLL, but preliminarily also in acute myeloid leukemia (AML) and acute lymphoblastic leukemia (ALL). BH3 mimetics bind to the hydrophobic groove of BCL2 inducing apoptosis. The precursor molecule of venetoclax, ABT-737 had a binding potential to BCL2, BCL-x_L_, and BCL-w, and showed activity in vitro. Navitoclax (ABT-263), an orally available derivate, showed promising efficacy in CLL patients [57]; however, strong inhibition of BCL-x_L_ induced a rapid decrease in circulating platelets and thrombocytopenia which arrested its clinical development.

**Fig. 2:**
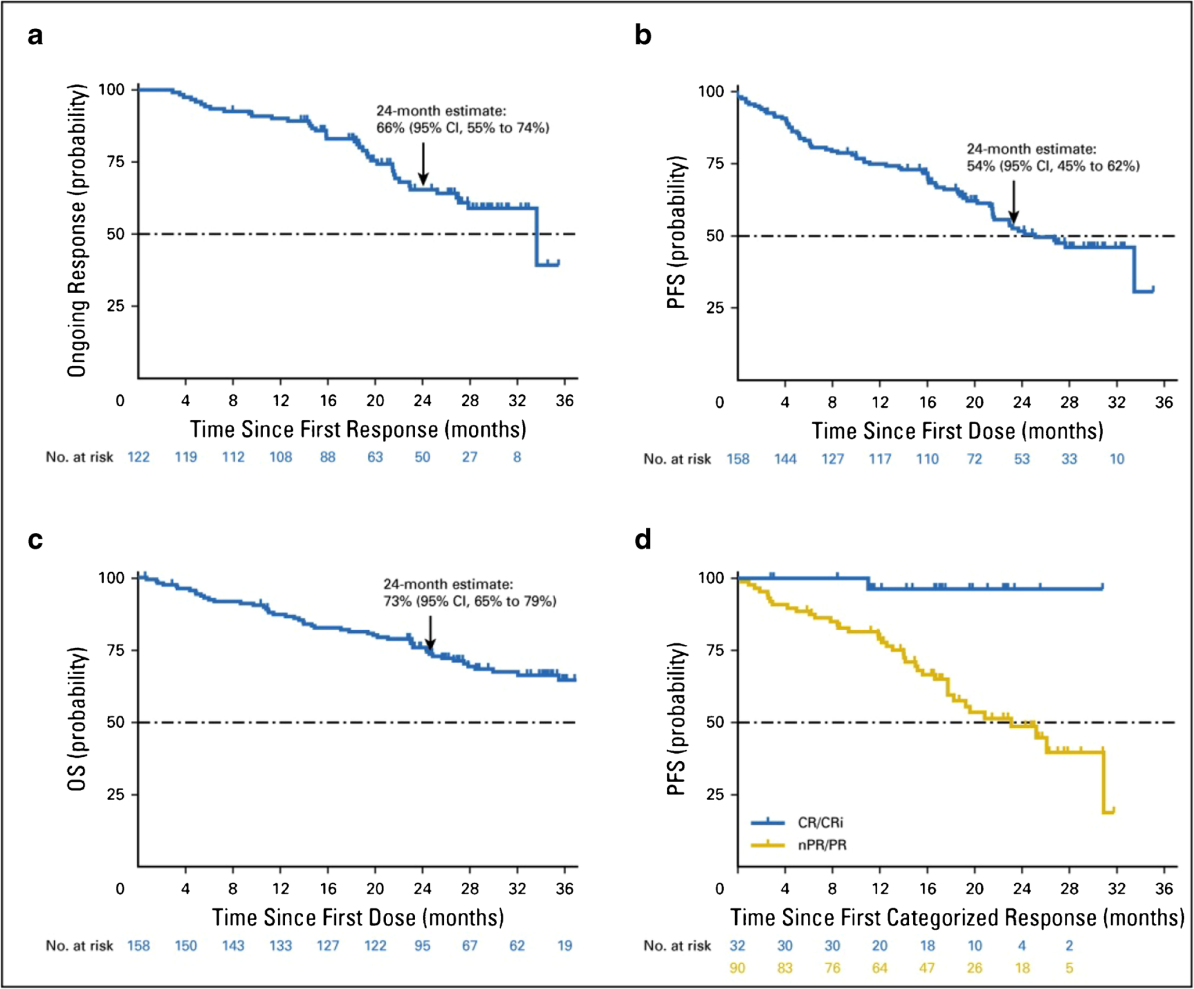
**a** Kaplan-Meier curve on venetoclax monotherapy. A total of 122 patients achieved response. PFS (**b**) and OS (**c**) for all 158 patients enrolled. **d** PFS of patients since the achievement of CR or CRi (yellow curve). PFS of 90 patients with nPR/PR (blue curve). Reprinted with permission. ©2018 American Society of Clinical Oncology. All rights reserved. Stilgenbauer S, Eichhorst B, Schetelig J, et al. Venetoclax for patients with chronic lymphocytic leukemia with 17p deletion: Results from the full population of a phase ii pivotal trial. *J Clin Oncol*. 2018;36:1973–1980 [59]

Venetoclax was approved by the FDA and EMA in December 2016 for patients with previously treated CLL with del(17p13) and patients failing B cell receptor signaling inhibitors (EMA only), or who carry a TP53 mutation and are refractory to chemoimmunotherapy and BCR inhibitors recruited in the phase II M13-982 and M14-032 trials [58•]. M13–982 was a pivotal phase II clinical trial, enrolling relapsed/refractory CLL patients with del(17p). In this multicenter open-label study, 158 patients were treated with venetoclax with a weekly dose ramp up from 20 to 400 mg over 4 weeks (20, 50, 100, 200, and 400 mg, due to the risk of tumor lysis syndrome (TLS), see below) which was continued until disease progression. At a median time of 26.6 months on study, 77% of all patients had achieved an ORR (122 of 158 patients). The 24-month estimate for ongoing response was 66% (95% CI, 55% to 74%, Fig.2a); the 24-month estimate of PFS and OS were 54% (95% CI, 45% to 62%, Fig. 2b), and 73% (95% CI, 65% to 79%, Fig. 2c), respectively. Estimated PFS of patients with complete remission (CR) or incomplete bone marrow recovery (CR_i_) or nodular partial remission (nPR) at 27.2 months was not reached (Fig. 2d) [59].

Responses of venetoclax treatment were durable, and a majority of the patients showed a reduction in absolute lymphocyte count, lymph node lesion diameter, and bone marrow infiltrate at a median of 0.3 months of treatment. Management of the tumor lysis syndrome occurred using prophylaxis in 6 patients; none of them reached clinical TLS. Hematologic adverse effects of higher grade (grade ≥ 3) were neutropenia (42%), anemia (25%), and thrombocytopenia (20%). The results of this pivotal trial led to FDA approval of venetoclax in April 2016 for the treatment of previously treated CLL patients with del(17p) [60•].

M14-032 recruited relapsed/refractory patients after previous treatment with ibrutinib or idelalisib. The ORR in the ibrutinib pretreated group was 65%, the 12-month PFS was 75%. In the idelalisib-pretreated group, the ORR was 67%, and an estimated 1-year progression-free survival of 79% was found [61]. Most common grade 3 or 4 adverse effects were neutropenia (51%), thrombocytopenia (29%), and anemia (29%). Of 91, 17 patients died because of progressive disease [61]. Importantly, venetoclax induced deep remissions with MRD negativity in contrast to treatment with BCR pathway inhibitors [62]. No incidence of tumor lysis syndrome (TLS) was observed.

Since the application of venetoclax induces a rapid reduction of tumor cells with abrupt onset within 6–8 h after dosing, TLS is a major risk issue in clinical care, owing to the high potency of the drug. Dimension of TLS, resulting in rapid cell death, is dependent on tumor mass [63, 64], comorbidities such as renal function, and treatment dose [63]. Quick release of metabolites into the blood stream destabilizes renal excretion, which causes hyperuricemia, hyperkalemia, hyperphosphatemia, and hypocalcemia, following acute renal failure and cardiac events of life-threatening potential. For this reason, patients in early treatment phase should be monitored closely for TLS. Prophylaxis of TLS including hydration, diuresis, monitoring of electrolytes, and prevention of hyperuricemia should be performed according to its severity and treatment following the guidelines on risk assessment and prophylaxis of TLS [65].

Despite high rates of durable remission in patients, continuous daily treatment with venetoclax induces secondary resistance. Venetoclax resistance was shown to be implicated with complex clonal shifts [66]. Using an in vitro genome-scale screen, Guièze et al. identified regulators of lymphoid transcription and cellular metabolism as resistance drivers. Confirmation in CLL patient cells with early progressive disease uncovered MCL-1 overexpression and AMPK signaling responsible for venetoclax resistance [66]. Whole exome sequencing of 8 CLL patients with dysfunctional TP53 and a median time of 15.4 months from initial treatment until progression uncovered heterogeneous clonal evolution under venetoclax treatment. Two patients developed a *BTG1* mutation; 3 patients had a homozygous deletion affecting *CDKN2A/B*, one *BRAF* mutation, and an amplification of CD274 were deciphered [67]. Very recently, another recurrent mutation was reported by comparison of paired pre-venetoclax and progression samples after long-term treatment. Seven of 15 patients carried a novel Gly101Val mutation in *BCL2*, which was selected at progression after 19 to 42 months, but not at the beginning of treatment [68]. The authors showed that Gly101Val reduces the affinity of BCL2 for venetoclax by 180-fold. In a second report, Tausch et al. identified the *BCL2*^Gly101Val^ mutation and an additional D103Y mutation in 3 of 4 venetoclax progressive patients [69]. These data demonstrate a selection of cell clones which acquire mutations under selection pressure of venetoclax. Especially, long-term treatment is prone to drive the evolution of resistant tumor cells. Clinical trials therefore aimed at time-limited, highly effective combination strategies to obtain deep remissions and to prevent secondary resistance generation.

The MURANO trial was the pivotal trial establishing time-limited therapy with venetoclax and led to the extension of the FDA and EMA approval of venetoclax and rituximab for all R/R CLL patients, regardless their del(17p) status. This phase III trial aimed to test efficacy and safety of the combination venetoclax and rituximab (VR) in order to avoid secondary resistance generation. In brief, 194 rel/ref. patients were treated 2 years with venetoclax and received 6 cycles of rituximab, compared with 6 cycles of bendamustine-rituximab (BR). At 24 months, PFS was estimated to be 84.9% compared with 36.3%, respectively. MRD negativity, OS, and ORR were impressively improved. After termination of venetoclax, follow-up at 3 years demonstrated an excellent benefit of VR against BR in regard to PFS (71.4% vs. 15.2%) with slightly enriched adverse effects (neutropenia 57.7% vs. 38.8%) in the VR group [70].

In May 2019, the FDA approved venetoclax for front line CLL treatment based on the CLL14 study [71]. Four hundred thirty-two patients with previously untreated CLL and coexisting medical conditions were randomized to venetoclax and obinutuzumab (Ven + G) or obinutuzumab + chlorambucil (GClb). After a follow-up of 24 months, the Kaplan-Meier estimate of the percentage of PFS was significantly higher in the Ven + G group than in the GClb group (88.2% vs. 64.1%); PFS was still significantly improved at 28.1 months in the Ven + G group (30 primary end-points counted vs. 77). Moreover, patients with TP53 deletion, mutations, or unmutated IGHV had a significant benefit as well. Adverse effects as grade 3 or 4 neutropenia or infections were comparable in both of the arms [71].

## Combination Trials of BCR Inhibitors and BCL2 Inhibitors

Venetoclax and ibrutinib have two different modes of action, and preclinical studies in human and mouse CLL cells demonstrated synergistic activity [72, 73]. Efficacy profiles of the two drugs behave also complementarily, since ibrutinib-dependent lymphocytosis leads to clearance of tumor cells from lymph nodes and mobilizes them into the peripheral blood. In contrast to ibrutinib, venetoclax-induced apoptosis has prominent efficacy in the blood and marrow. Moreover, venetoclax is able to induce MRD negativity, which rarely occurred with ibrutinib treatment. Thus, the CAPTIVATE trial analyzed the combination of ibrutinib and venetoclax in 163 treatment naïve CLL. In the combination arm, patients received single-agent ibrutinib for the first 3 cycles (cycle = 28 days) followed by an ibrutinib plus venetoclax combination for at least 12 cycles. After MRD negativity, ibrutinib was continued daily. Early analysis shows a CR rate of 100% as well as MRD negativity of 82% [74].

Following a similar strategy, the ongoing phase II CLARITY trial aims to determine therapeutic activity and safety of this combination with the intention to stop therapy. An interim result demonstrated that the primary endpoint, MRD negativity, was achieved in 28/53 (53%) patients in PB and 19/28 (36%) in bone marrow. Impressively, 89% patients responded, and CR rate was 51%, ongoing with high tolerance and acceptable adverse events [75].

Another phase II study of combined ibrutinib and venetoclax involved 80 naïve high-risk and older patients. Patients carried at least a high-risk feature (del(*TP53*) or *TP53*mut, del(11q), or unmutated IGHV). Impressive responses across all subgroups in the subset of patients who completed 12 cycles of treatment with 88% of complete remission and 61% remission with undetectable MRD [76].

Further trials which are still ongoing are the NCT02756897 (combined treatment of ibrutinib and venetoclax), NCT03226301 and NCT03045328 (combined treatment of ibrutinib and venetoclax in R/R patients), NCT03128879 (high risk CLL), and NCT03513562 (patients with ibrutinib-resistant mutations).

Further development aiming at deep responses are triple therapies with ibrutinib, venetoclax, and obinutuzumab (NCT02427451, NCT02758665/CLL2-GIVe, and NCT02950051/CLL13).

NCT02427451 was reported in 2018 by Rogers et al. In the phase 1b study, a small cohort of 12 R/R patients received 4 doses of obinutuzumab up to 8 courses. In parallel, they were treated with ibrutinib daily starting on course 2 and venetoclax, starting at course 3 for a total of 14 courses. To minimize risk of TLS, ibrutinib and venetoclax were introduced sequentially. The overall response rate was 92% with 6 patients being MRD negative at the end of treatment. Notably, no clinical or laboratory TLS occurred. Of the patients, 33% sustained grade ≥ 3 neutropenia, which is comparable with single treatment of venetoclax [77].

The phase 2 CLL2 BAG trial aimed to first reduce tumor cells using bendamustine followed by maintenance therapy by obinutuzumab and venetoclax up to 24 months. A total of 66 patients were enrolled and treated with the sequential triple T concept. First, the 63 patients (34 treatment naïve and 29 R/R) received bendamustine for the debulking of tumor cells. After two 28-day cycles, maintenance therapy with obinutuzumab and venetoclax in a weekly dose escalation was proceeded. To date, most common adverse effects were neutropenia (44%), infections (14%), and thrombocytopenia (9%) [78].

The primary objective of the multicenter phase II CLL2-GIVe trial aims to evaluate the efficacy of ibrutinib and venetoclax and obinutuzumab in physically fit or unfit, previously untreated patients with del(17p) or TP53 mutation [79] Currently, all 41 high-risk patients have been recruited, and the primary endpoint (CR rate after 12 cycles of treatment) will be available in Q2 2020.

The phase III CLL13 trial (GAIA), an international four arm study for physically fit patients, is testing chemotherapy-free frontline therapy for previously untreated patients without del(17p). The trial is fully enrolled and tested with standard chemotherapy (FCR/BR), venetoclax plus rituximab (RVe), venetoclax plus obinutuzumab (GVe) and venetoclax plus ibrutinib, and obinutuzumab (GIVe) with MRD and PFS as co-primary endpoints [80].

## Conclusion

Ibrutinib and venetoclax monotherapy have been initially licensed based on remarkable single-agent efficacy and favorable tolerability profile, including chemo-refractory and genetic high-risk subgroups of CLL. However, resistance due to specific mechanisms (e.g., *BTK*, *PLCg2*, and *BCL2* mutations) is emerging indicating the need to develop time-limited combinations. Approved combination treatments such as venetoclax or ibrutinib with obinutuzumab show high response rates and long remission durations. It is possible that these strategies may lead to long-term remission and potential cure of CLL. Comparison of sequential treatment with combinations and discontinuation of therapy are important aspects which need to be investigated.

Based on the high rate of deep responses and long remission durations combined with good tolerability, venetoclax-based combinations with BTK inhibitors or triple combinations are very likely the future of CLL treatment; however, the approaches are still experimental and require more clinical data before adapted in general practice.
